# Accurate surgical navigation with real-time tumor tracking in cancer surgery

**DOI:** 10.1038/s41698-020-0115-0

**Published:** 2020-04-08

**Authors:** Esther N. D. Kok, Roeland Eppenga, Koert F. D. Kuhlmann, Harald C. Groen, Ruben van Veen, Jolanda M. van Dieren, Thomas R. de Wijkerslooth, Monique van Leerdam, Doenja M. J. Lambregts, Wouter J. Heerink, Nikie J. Hoetjes, Oleksandra Ivashchenko, Geerard L. Beets, Arend G. J. Aalbers, Jasper Nijkamp, Theo J. M. Ruers

**Affiliations:** 1 Department of Surgical Oncology, The Netherlands Cancer Institute, Amsterdam, The Netherlands; 2 Department of Gastrointestinal Oncology, The Netherlands Cancer Institute, Amsterdam, The Netherlands; 3 Department of Radiology, The Netherlands Cancer Institute, Amsterdam, The Netherlands; 4 Faculty TNW, Group Nanobiophysics, Twente University, Enschede, 7522 NB The Netherlands

**Keywords:** Surgical oncology, Rectal cancer

## Abstract

In the past decades, image-guided surgery has evolved rapidly. In procedures with a relatively fixed target area, like neurosurgery and orthopedics, this has led to improved patient outcomes. In cancer surgery, intraoperative guidance could be of great benefit to secure radical resection margins since residual disease is associated with local recurrence and poor survival. However, most tumor lesions are mobile with a constantly changing position. Here, we present an innovative technique for real-time tumor tracking in cancer surgery. In this study, we evaluated the feasibility of real-time tumor tracking during rectal cancer surgery. The application of real-time tumor tracking using an intraoperative navigation system is feasible and safe with a high median target registration accuracy of 3 mm. This technique allows oncological surgeons to obtain real-time accurate information on tumor location, as well as critical anatomical information. This study demonstrates that real-time tumor tracking is feasible and could potentially decrease positive resection margins and improve patient outcome.

## Introduction

In the past two decades, image-guided surgery has evolved rapidly. The technique enables real-time visualization of surgical instrumentation with respect to intraoperative anatomy based on preoperative imaging. A prerequisite of the current surgical navigation systems is a fixed target area since registration of the preoperative images with the intraoperative anatomy is based on this rigid, fixed position.

In view of this condition, the technique was initially developed in neurosurgery, in which the soft-tissue component has a fixed position with respect to the bone. The main goal was to improve precise targeting of the tumor margins and improve the safety of the surgical procedures. Image-guided neurosurgery evolved from frame-based stereotaxic to frameless navigation^1^. Nowadays, multiple studies showed that image-guided neurosurgery is feasible and improves the extent of resection of malignant brain tumors^2,3^. Additionally, the technique expanded to orthopedic and head and neck surgery where it showed to be of added value with improved accurate placement of surgical implants^4,5^.

Besides these applications, intraoperative guidance could be of great benefit to many other surgical oncology procedures not confined to a rigid surrounding. During such procedures, surgeons rely on palpation and visual inspection to distinguish tumorous from healthy tissue. This can be challenging and may result in incomplete removal of the tumor tissue, associated with local recurrence and poor overall survival^6–9^. To make navigation useful under such circumstances and improve surgical outcomes, the technique has to account for the changing position of the tumor by real-time tumor tracking during surgery.

In this study, we present a new technique that brings surgical navigation to the next stage of development and make it suitable for a wide range of applications in surgical oncology. To this end, we developed an innovative technology that allows for real-time tumor tracking during surgery. Here, we report on the feasibility and accuracy of this innovative technique in patients undergoing rectal surgery. In rectal cancer surgery, total mesorectal excision (TME) is the golden standard. TME can be challenging with limited visualization and surgical access due to the anatomical location of the tumor. Because of this, the amount of tumor positive resection margins still remains 10–15%^10,11^. Therefore, better intraoperative guidance would be of great benefit to secure radical resection margins.

The study was performed in two phases. In the first phase (feasibility phase), feasibility and safety of the study workflow were investigated, the possibility of real-time tumor tracking with the navigation system was assessed and possible improvements of the clinical workflow were identified. In the second phase (test phase), the ultimate accuracy of the real-time tracking was measured.

## Results

### Feasibility phase

**Fig. 1:**
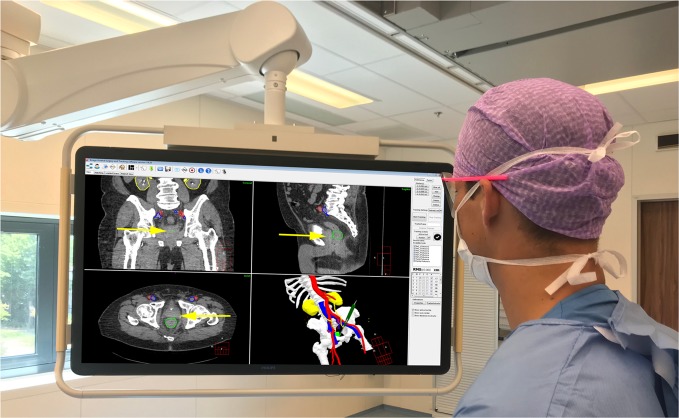
Surgical navigation user interface during surgery showing the planning CT with segmentations (top-left corner: coronal view; top-right corner: sagittal view; lower-left corner: axial view) and 3D model (lower-right corner). Visible segmentations: bones (white), arteries (red), veins (blue), ureters/kidneys (yellow), and tumor (green). The visible slice of the CT scans is based on the location of the tip of the surgical pointer which is highlighted with yellow arrows.

**Fig. 2:**
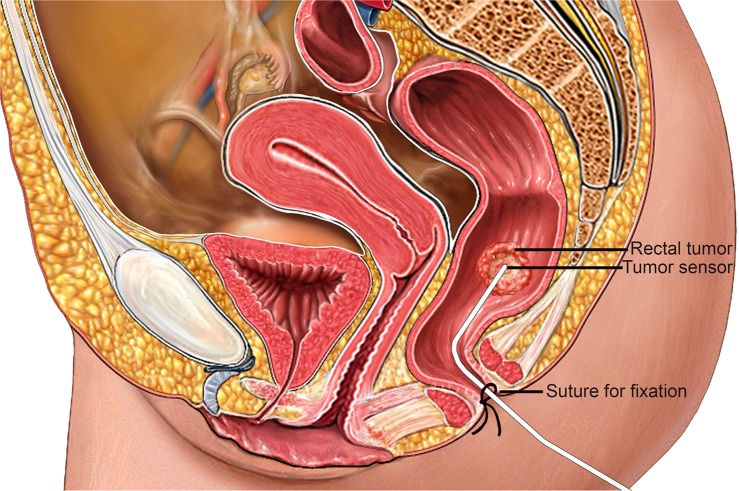
Schematic overview of in vivo tumor sensor with the tip positioned by the surgeon at the rectal tumor. The sensor is fixated with sutures in the perineal area (Alamy Stock Photo (https://www.alamy.com/search.html?qt=Sagittal%20View%20of%20a%20Female%20Pelvis&imgt=0), used and edited with permission).

**Fig. 3:**
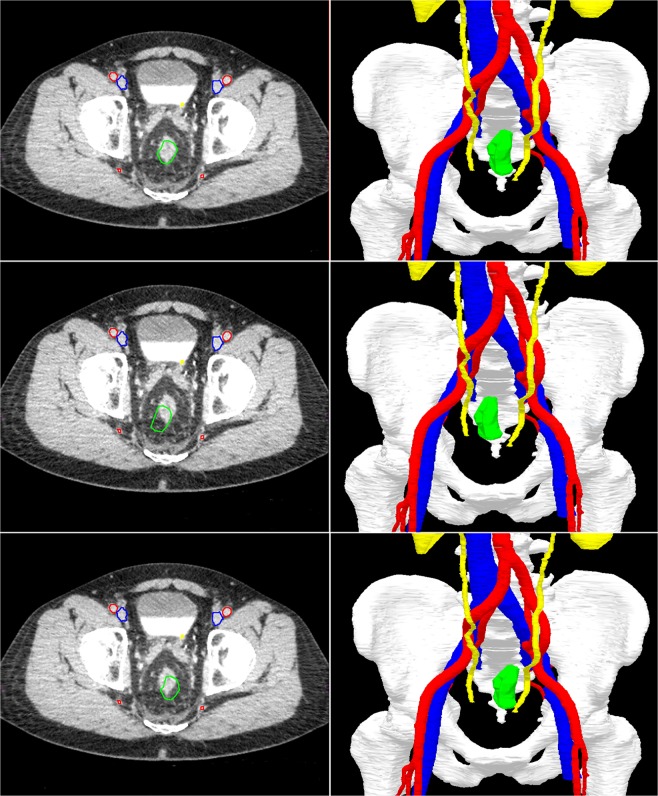
An example to illustrate the real-time tumor tracking by the navigation system. The top two images show the position of the rectal tumor in rest. In the lower four images, the rectum is moved to the right and left. Note that the green segmentation area follows the real-time tumor position in the navigation images on the right, while the CT scan on the left (still) shows the original position of the tumor. Visible segmentation: bones (white), arteries (red), veins (blue), ureters/kidneys (yellow), and tumor (green).

Between November 2016 and March 2018, 15 patients with histological proven rectal cancer were included (Table 1). In all patients, a patient-specific three-dimensional (3D) model was created based on preoperative imaging. The time to segment the preoperative images took around 2 h. A computer screen in the OR showed the 3D model, three orthogonal views (axial, sagittal, and coronal) of the preoperative images, and the tracked tumor and pointer position during surgery (Fig. 1). A schematic overview of the position of the tumor sensor that adjusted for real-time tumor movements is shown in Fig. 2 and an example of the tracked tumor movement in Fig. 3. In the feasibility phase, 11 patients (73.3%) successfully completed the entire investigational workflow and received rectal surgery with the navigation technique. In four patients (26.7%) the tumor sensor was damaged during placement and discontinued working. Therefore, the sensor was inserted in a silicone round surgical wound drain (3.3 mm) for protection before placement into the rectum in the subsequent patients (starting at patient 9). During the feasibility phase, tumor registration was challenging due to the low soft-tissue contrast on the CBCT. Visual evaluation of the registration during surgery, when the patient was in Trendelenburg position, showed that manual correction of the system was necessary in four patients with an average of 3.8 mm in the left–right direction, 9.4 mm caudal–cranial direction, and 5.5 mm in anterior–posterior direction. In one patient, the intratumoral fiducial was lost after placement. Registration was based on the remaining two fiducials in this patient. No navigation-related complications were observed. Treatment-related characteristics are shown in Table 2. Placement of the patient trackers, positioning the tumor sensor, and obtaining the intraoperative CBCT scan added on average 31 min (range: 21–46) to the total surgical procedure time.

**Table 1:** Patient characteristics.

Characteristics	Feasibility phase, *n* = 15	Test phase, *n* = 16
Sex		
Male	10 (66.7)	11 (68.7)
Female	5 (33.3)	5 (31.3)
Median age (year) (min–max)	62.0 (39–78)	58.0 (37–85)
Clinical tumor and nodal stage^a^		
cT2N0	0 (0.0)	1 (6.3)
cT2N+	2 (13.3)	2 (12.5)
cT3N0	6 (40.0)	8 (50.0)
cT3N+	5 (33.3)	5 (31.3)
cT4N0	1 (6.7)	0 (0.0)
cT4N+	1 (6.7)	0 (0.0)
Median distance between anorectal verge and tumor (cm)	3.0 (0–9)	3.0 (0–6)
Neoadjuvant treatment		
Radiotherapy (5 × 5 Gy)	3 (20.0)	1 (6.3)
Radiotherapy (5 × 5 Gy) + chemotherapy	5 (33.3)	4 (25.0)
Chemoradiation (25 × 2 Gy + capecitabine)	7 (46.7)	9 (56.3)
Chemoradiation + chemotherapy	0 (0)	1 (6.3)
Chemoradiation and contact radiotherapy	0 (0)	1 (6.3)

^a^Clinical tumor and nodal stage at diagnosis.

**Table 2:** Treatment characteristics.

Characteristics	Feasibility phase, *n* = 15	Test phase, *n* = 16
Fiducial markers	8 (53.3)	16 (100.0)
Type of surgery		
Open APR	4 (26.7)	4 (25.0)
Lap. APR	3 (20.0)	4 (25.0)
Open LAR	5 (33.3)	3 (18.8)
Lap. LAR	3 (20.0)	3 (18.8)
No navigated surgery performed	0 (0)	2 (12.5)
Surgery with navigation	11 (73.3)	14 (87.5)
Technical navigation errors		
Tumor sensor-related	4 (26.7)	0 (0)
Registration-related	0 (0)	1 (6.3)
Study-related severe adverse events^a^	0 (0)	1 (6.3)
Intraoperative complications^b^		
Navigation related	0 (0)	0 (0)
Non-navigation related	0 (0)	1 (6.3)
Median extra time needed (min–max)	31 (21–46)	21 (15–30)

^a^One patient suffered from an intra-abdominal infection possibly related to the fiducial placement.

^b^One patient experienced iatrogenic injury of the ureter, not caused or related to the navigation system, and required reimplantation of the ureter in the bladder.

### Test phase

Since tumor registration in the feasibility phase was challenging, patients received in the test phase fiducial markers close to the rectal tumor before the planning CT scan, at least 1 week before surgery. In the OR, registration of the tumor was performed based on the fiducial markers which allowed for a more accurate intraoperative tumor registration and tracking.

During the test phase, the definite protocol was evaluated in 16 patients (Table 1). Of these patients, 14 patients received rectal surgery with the navigation system. In two patients, navigation was not used during surgery. In the first patient, rectal surgery was canceled due to unexpected progression of metastatic disease. The second patient suffered from an intra-abdominal infection possibly related to the fiducial placement and underwent early resection without navigation. Treatment-related characteristics are shown in Table 2. During surgery with the patient in the Trendelenburg position, adjustment of the registration was performed in six patients. The average correction was 2.3 mm in the left–right direction, 13.0 mm caudal–cranial direction, and 3.8 mm in anterior–posterior direction. In one patient, the intratumoral fiducial was lost after placement, resulting in a tumor registration based on the remaining two fiducials in this patient.

In the test phase, placement of the patient trackers, positioning of the tumor sensor, and obtaining the intraoperative CBCT scan added on average 21 min (range: 15–30) to the total surgical procedure time. Real-time tracking of the rectal tumor was feasible in 13 of the 14 patients (92.9%). In one patient, despite manual correction during the visual evaluation, no accurate registration could be achieved and the registration error remained >2 cm. Therefore, no interpretable navigation data could be collected. No navigation-related complications were observed during surgery.

Accuracy of the navigation setup was determined by correlation of the position of the proximal tumor margin, as determined during navigation and marked by surgical clips, with the position of the proximal tumor margin as determined on the pathology specimen. Accuracy measurements could be achieved in 11 patients (84.6%) of the 13 patients with navigation data receiving rectal surgery. In one patient pathological correlation of tumor margins was not possible due to a complete pathological response (T0N0) after neoadjuvant treatment and in one other patient because the surgical clips to mark the proximal tumor margin during navigation fell off the rectal resection specimen during surgical removal. The median absolute distance between the surgical clips and the proximal tumor border at histopathology was 3 mm (IQR: 2–10 mm).

## Discussion

Image-guided surgical navigation has become widespread in head and neck surgery, neurosurgery, and orthopedics. In these fields, the technique is feasible due to the most rigid, bone-defined target area. To make surgical navigation a useful technique for cancer surgery in general, where tumor lesions often shift during surgery, real-time tumor tracking is necessary. In this article, we present a navigation system that fulfills this need and performs real-time tumor tracking during the course of surgery. The system was tested in rectal cancer surgery in both open and laparoscopic setting.

This prospective study shows that real-time tumor tracking with surgical navigation is feasible, safe, and accurate. During the feasibility phase, improvements in the workflow were made to enhance the practicality, speed, and accuracy of the tumor registration. After optimization of the workflow, a high median accuracy of 3 mm was obtained for surgical navigation in the test phase of the study. No navigation-related complications were observed during surgery and the total extra time decreased with one-third towards the end of the study to 21 min. The accuracy obtained is clinically relevant and warrants further clinical implementation.

In the past years, some limited case series evaluated the feasibility of surgical navigation in pelvic surgery. The group of Atallah et al.^12–14^ published three articles, including five patients in total, on stereotactic navigation using an optical tracking system for transanal minimal invasive surgery—TME (TAMIS-TME). In all three studies, they concluded that stereotactic navigation for TAMIS-TME was feasible and safe with an accuracy of around 4 mm. However, the accuracy of the system did not assume tumor or patient movement during the procedure as the trackers were mounted on to the bedrail. Kwak et al.^15^ also performed navigation in pelvic surgery in two patients, stating that surgical navigation was feasible with acceptable accuracy of the target. Although both groups showed encouraging results, no real-time tracking of the rectal tumor was performed and the rectal tumor was assumed to be fixed in the same position during the procedure.

The main challenge of the current study was to track tumor movement in real time in every possible direction. This could be accomplished by introducing a six degree of freedom sensor close to the tumor lesion. The current results confirm earlier results from an ex vivo rectum study by Wagner et al.^16^. They developed a realistic rectum phantom to test motion and deformation using a tracking sensor. Multiple models were created to translate sensor movement to rectum wall deformations. In the most complex model, 33 parameters were taken into account which resulted in a target registration error of 2.9 ± 1.4 mm. Our results are in accordance with the results obtained in these phantom experiments.

A limiting factor for the accuracy measurements in the current in vivo application for rectal cancer may be that all patients in the current study were operated after neoadjuvant radiotherapy with a long interval to surgery. This makes interpretation of the preoperative imaging more challenging. Magnetic resonance imaging (MRI) is the most advanced staging technique in rectal cancer and routinely used for preoperative staging and restaging^17,18^. Unfortunately, interpretation of MRI is known to be less reliable when patients are pretreated with chemoradiation or radiotherapy. Multiple studies evaluated MRI for restaging and showed overall a low sensitivity, which is mainly caused by difficulties to differentiate areas of vital residual tumor within radiation-induced fibrosis^19,20^. In this study, all segmentations of the tumor and other critical structures were validated by an experienced radiologist or a rectal cancer MRI expert and the operating surgeon. Despite this challenging task, a median navigation accuracy of 3 mm was established.

Besides the interpretation of the MRI, some other factors may have influenced the accuracy. For example, the obtained accuracy may be dependent on the positioning and registration of the fiducials. Fiducial placement was performed using a sigmoidoscopy in combination with endoscopic ultrasonography (EUS) but in smaller limited-volume tumors, adequate positioning of the fiducials on the proximal and distal tumor was challenging. Furthermore, it remains uncertain if the position of the fiducials was indeed rigid with respect to the tumor. Another factor that might have influenced the accuracy is the pathological processing of the rectal specimen. The distance between the surgical clips and the proximal tumor border was measured manually by the pathologist and reproduction of the axial tumor plane was in some patients complex.

Manual correction of the registration was performed in 10 out of 29 patients. When corrections were applied, these were almost exclusively related to the caudal–cranial axis (9 out of 10). A possible explanation for the inaccuracy in this direction may be found in the way patients were tracked during surgery. Patient trackers were taped to the skin close to the bone to correct for patient movement with respect to the table and field generator. However, some movement of the skin and the patient trackers with respect to the pelvis may be inevitable, especially when the patient is moved to the Trendelenburg position during surgery. Obtaining the CBCT scan after the patient is positioned to Trendelenburg is unfortunately not possible since a security lock in the CBCT scanner prevents scanning when the bed is not positioned straight. However, this slight movement could easily be corrected for by visual correction of the registration after the patient was positioned in Trendelenburg.

During the study, adjustments were performed improving the practicality and the accuracy of the tumor registration. However, positioning and securing the tumor sensor against the rectal tumor remains inconvenient and adds additional time to the anesthesia. A solution may be found in the use of wireless EM-tracked transponders^21–23^. These wireless transponders could be implanted near the tumor the day before surgery when the patient would be admitted to the hospital, and their position could be visualized by CT the same day. During the OR no additional imaging would be necessary, and any shift of tumor location could simply be tracked in real time by tracking the responder position.

In conclusion, this study shows that real-time tumor tracking for cancer surgery is feasible and safe. The accuracy of the system is high, which justifies broader clinical implementation. Further studies will be initiated to improve the workflow and prove clinical benefit.

## Methods

### Clinical trial design and patient inclusion

This prospective feasibility study was conducted in The Netherlands Cancer Institute from November 2016 to August 2019. The study was approved by the institutional review board in August 2016 (NL57251.031.16/N16TRS). The study was conducted according to the principles of the Declaration of Helsinki and in accordance with the Medical Research Involving Human Subjects Act (WMO). Inclusion criteria were patients with rectal cancer scheduled for open or laparoscopic low anterior resection (LAR) or abdominoperineal resection (APR), patient age ≥18 years, tumor distance from anal verge <10 cm (based on preoperative imaging). Exclusion criteria were contraindications for intravenous contrast administration (allergy or severely impaired kidney function), cardiac pacemaker and metal implants in the pelvic area causing artifacts on the preoperative imaging. All participating patients gave written informed consent. The study was registered at www.trialregister.nl (identifier: NL7666).

The study was performed in two phases. In the first phase (feasibility phase), 15 patients were included. The feasibility and safety of the study workflow were investigated, the possibility of real-time tumor tracking with the navigation system was assessed, and possible improvements of the study protocol were identified. After optimization of the study protocol, the second phase (test phase) was initiated. In this test phase, 16 patients were included and the accuracy of the real-time tracking was determined.

### Navigation system

An NDI Aurora V2 electromagnetic (EM) tracking system (Northern Digital Inc., Waterloo, Ontario, Canada) was used to link preoperative imaging data to the intraoperative patient setup. Patients were positioned on an operating table including a tabletop field generator (TTFG) with an oval EM field of 42 × 60 × 60 cm. Patient trackers (Philips Traxtal/percunav, Philips, Best, The Netherlands) with EM sensors were used to determine the position of the patient during surgery and a tracking sensor was placed against the tumor to adjust for real-time tumor movements. Within a hybrid operating room, a cone-beam computed tomography (CBCT) scan (Philips Allura FD20 XperCT; Philips) was performed after sensor placement but before the start of surgery. Acquired images were registered to the preoperative diagnostic planning CT scan using the in-house developed navigation software. During surgery, an EM-tracked surgical pointer (NDI) was available to validate the accuracy of the registration.

### Workflow in feasibility phase

A preoperative diagnostic multiphase contrast-enhanced abdominal CT scan (planning CT with early arterial and excretion phase)—acquired for the purpose of this study one day before surgery—and diagnostic MRI scans were used to create a patient-specific 3D model. The 3D model consisting of bones, arteries, veins, ureters, rectum, and tumor was created using semi-automated in-house developed segmentation software. Tumor delineation was supervised by an experienced radiologist or study investigator with extensive experience in rectal cancer MRI. Resulting 3D models were evaluated by the operating surgeon before surgery.

In the operation room (OR), three patients trackers (PercuNav Patient Tracker, Philips) were taped to the skin of the patient. One patient tracker was positioned at the level of the anterior superior iliac spine and the remaining two left and right of the spine in the lumbar curvature. Under anesthesia, a rectal examination was performed by the surgeon. With a proctoscope, a wired 6 degree of freedom EM-sensor (Flextube, Northern Digital Inc., Waterloo, Ontario, Canada) was fixated on the tumor using surgical glue. The patient was placed in surgical position and a CBCT image of the pelvic area including the patient trackers was acquired.

Because of the high mobility of the rectum, accurate registration of the intraoperative CBCT scan to the planning CT consisted of two steps: (I) rigid registration of the (semi) rigid parts of the model (i.e., bones and main vessels) and (II) deformable registration of the tumor. First, the bony anatomy on the intraoperative CBCT scan was linked to the 3D model by rigid registration. Subsequently, maintaining the registration of the bones and semi-rigid anatomy unchanged, a separate deformable tumor registration was performed based on gray values. The resulting navigation information consisted of the rigid anatomy of the pelvis plus a non-rigid representation of the tumor. The tumor sensor and pointer were automatically real-time tracked by the system and visible on a computer screen in the OR^24^. On this screen, the 3D model and three orthogonal views (axial, sagittal, and coronal) of the obtained images were shown (Fig. 1). An example of the tracked tumor movement during surgery is shown in Fig. 3.

At the start of surgery, the actual position of the pointer was visually compared to the pointer position in the navigation system. In case of a visual inaccuracy of ≥2 mm, the registration was adjusted accordingly. During further surgery, visual evaluation of the registration accuracy was performed by placing the tip of the pointer at anatomic landmarks like the sacral promontory, ureters, or the common iliac artery bifurcation. If necessary, manual correction was performed on both the rigid anatomy as well as the tumor.

### Workflow in test phase

For the test phase, the clinical workflow was changed with regard to tumor sensor placement and registration of the tumor.

To secure protection for breakage and guarantee a more stable tumor sensor position, the sensor was inserted in a round silicone surgical wound drain (3.3 mm) before placement into the rectum. After positioning the tip of the sensor against the rectal tumor, the silicone wound drain was fixated to the skin in the perineal area with stwo sutures.

In the feasibility phase, tumor registration was challenging due to the low soft-tissue contrast on the CBCT. In the test phase, to improve tumor registration, patients received fiducial markers close to the rectal tumor before the diagnostic CT scan, at least 1 week before surgery. These markers were used to facilitate tumor registration between the low soft-tissue contrast CBCT images and the high soft-tissue contrast of the planning CT. Fiducial placement in the rectum is often used for tumor localization during radiotherapy and has been reported as safe with excellent visibility on CT^25–27^. Patients received an enema before the procedure. Using flexible sigmoidoscopy in combination with EUS, an experienced gastroenterologist inserted three fiducials through the bowel wall in the mesorectal fat (one on the proximal and distal tumor border and one halfway the tumor). The locations of the fiducials were assumed to be rigid with respect to the tumor. Two types of fiducial markers were used: Gold Anchor GA150-20 0.28 mm × 20 mm unfolded length (Naslund Medical AB, Huddinge, Sweden) and Visicoil 0.5 mm × 5 mm (Core Oncology, Santa Barbara, California). The Gold Anchors were folded when inserted. Preoperative fiducial placement by the gastroenterologist took 20 min on average.

In the OR, after the bone to bone registration, registration of the tumor was performed based on the fiducial markers. This allowed for a more accurate intraoperative tumor registration and tracking.

### Accuracy measurements

In the test phase, an axial plane was added to the 3D model to accentuate the proximal tumor border. During surgery, the surgeon used the navigation interface to place two titanium surgical clips on the surface of the rectum in the plane of the proximal tumor border as given by the 3D model. After the specimen was resected, it was taken to the pathology department and processed according to standard protocol^28^. The distance between the surgical clips and the proximal tumor border was macroscopically reported and verified with histopathology.

If the surgeon was not able to reach the proximal tumor border (as indicated by the navigation system) with the surgical instruments due to narrow pelvic space, surgical clips were placed as close as possible cranial of the axial plane. The difference between the distance of the clips relative to the proximal tumor border measured by the navigation system and the pathology measurements was used as the accuracy value.

### Outcome parameters

The main goal of the feasibility phase was to assess if real-time tracking of the rectal tumor using the in-house developed EM navigation system was feasible and safe. Feasibility was defined as successful completion of the whole investigational workflow resulting in continuous delivery of interpretable navigation data for rectal surgery. In addition, the amount of extra time needed to use the system during surgery was evaluated.

The main goal of the test phase was to evaluate the accuracy of the system, which was validated by the distance (in mm) between the intraoperatively placed surgical clips, illustrating the proximal tumor border according to the 3D model, and the proximal tumor border as determined by pathology. In this phase, the amount of extra time was also reported. All analyses were performed in SPSS version 24.0® (IBM Corporation, Armonk, NY, USA).

## Supplementary information

Nr-reporting-summary

## Data Availability

The data used for the current study are available from the corresponding author (E.N.D.K.) upon reasonable request and through collaborative investigations.

## Code availability

The software used for the current study are available from the corresponding author (E.N.D.K.) upon reasonable request and through collaborative investigations.
